# Morphological features of microglial cells in the hippocampal dentate gyrus of Gunn rat: a possible schizophrenia animal model

**DOI:** 10.1186/1742-2094-9-56

**Published:** 2012-03-16

**Authors:** Kristian Liaury, Tsuyoshi Miyaoka, Toshiko Tsumori, Motohide Furuya, Rei Wake, Masa Ieda, Keiko Tsuchie, Michiyo Taki, Kotomi Ishihara, Andi Jayalangkara Tanra, Jun Horiguchi

**Affiliations:** 1 Department of Psychiatry, Shimane University Faculty of Medicine, 89-1 Enya-cho, Izumo 693-8501, Japan; 2 Department of Anatomy and Morphological Neuroscience, Shimane University Faculty of Medicine, 89-1 Enya-cho, Izumo 693-8501, Japan; 3 Department of Psychiatry, Hasanuddin University Faculty of Medicine, Makassar, Jl. Perintis Kemerdekaan Km. 10, Makassar 90245, South Sulawesi, Indonesia

**Keywords:** Microglial cells, Dentate gyrus, Gunn rats, Schizophrenia

## Abstract

**Background:**

Schizophrenia is a debilitating and complex mental disorder whose exact etiology remains unknown. There is growing amount of evidence of a relationship between neuroinflammation, as demonstrated by microglial activation, and schizophrenia. Our previous studies have proposed that hyperbilirubinemia plays a role in the pathophysiology of schizophrenia. Furthermore, we suggested the Gunn rat, an animal model of bilirubin encephalopathy, as a possible animal model of schizophrenia. However, the effects of unconjugated bilirubin on microglia, the resident immune cell of the CNS, in Gunn rats have never been investigated. In the present study, we examined how microglial cells respond to bilirubin toxicity in adult Gunn rats.

**Methods:**

Using immunohistochemical techniques, we compared the distribution, morphology, and ultrastructural features of microglial cells in Gunn rats with Wistar rats as a normal control. We also determined the ratio of activated and resting microglia and observed microglia-neuron interactions. We characterized the microglial cells in the hippocampal dentate gyrus.

**Results:**

We found that microglial cells showed activated morphology in the hilus, subgranular zone, and granular layer of the Gunn rat hippocampal dentate gyrus. There was no significant difference between cell numbers between in Gunn rats and controls. However, there was significant difference in the area of CD11b expression in the hippocampal dentate gyrus. Ultrastructurally, microglial cells often contained rich enlarged rich organelles in the cytoplasm and showed some phagocytic function.

**Conclusions:**

We propose that activation of microglia could be an important causal factor of the behavioral abnormalities and neuropathological changes in Gunn rats. These findings may provide basic information for further assessment of the Gunn rat as an animal model of schizophrenia.

## Introduction

Schizophrenia is a complex and debilitating mental disorder with a prevalence of approximately 1% worldwide. In addition to severely disrupting the life of patients and their families, schizophrenia imposes a great cost on society in terms of productivity loss and treatment-related expense. Schizophrenia is still a major challenge in psychiatry, in part because the exact etiology remains unknown [1,2].

Recent genome-wide studies in schizophrenia have shown the association of schizophrenia with markers in the major histocompatibility complex (MHC) region and suggest immune system involvement in schizophrenia [3]. An accumulating body of evidence point to the significance of neuroinflammation and immunogenetics in schizophrenia, characterized by an increased serum concentration of several proinflammatory cytokines [4]. Peripheral inflammatory responses in schizophrenia have also been linked to aberrations in circulating monocytes and T cells [5]. Interestingly, microglial activation or increased microglial cellular density has also been suggested by postmortem studies, at least in subpopulations of individuals with schizophrenia [6,7]. Moreover, a proinflammatory immune state influences the glutamatergic neurotransmission indirectly by bits effects on the tryptophan/kynurenine metabolism. The immune response in schizophrenia seems to be associated with the activation of the enzyme indoleamine 2,3-dioxygenase (IDO) and imbalance in tryptophan/kynurenine metabolism resulting in increased production of kynurenic acid in the brain. This is associated with an imbalance in the glutamatergic neurotransmission, leading to an *N*-methyl-D-aspartate (NMDA) antagonism in schizophrenia [8,9].

Previous studies showed a link between hyperbilirubinemia and schizophrenia. Schizophrenia patients have a significantly higher frequency of hyperbilirubinemia relative to patients with other psychiatric disorders and the general healthy population [10-12]. Moreover, in brain imaging studies, we found severer brain metabolism abnormalities in patients with schizophrenia associated with idiopathic unconjugated hyperbilirubinemia (Gilbert's syndrome (GS)) compared to both schizophrenia patients without GS and normal controls [13-16]. Based on these facts, we propose that hyperbilirubinemia may play a role in the pathophysiology of schizophrenia.

Hyperbilirubinemia is a common condition in the neonatal period that results from decreased erythrocyte survival and defective hepatic clearance of unconjugated bilirubin (UCB). Accumulation of UCB in the central nervous system (CNS) is the major cause of transient bilirubin encephalopathy and of a more critical condition, called 'kernicterus', which can result in a newborn infant's death [17-20]. Additionally, neonatal hyperbilirubinemia might be a vulnerability factor for later mental disorder [21].

Our previous study showed behavioral abnormalities, deficits in prepulse inhibition (PPI), and neuropathological changes in Gunn rats that are similar to the characteristics of schizophrenia [22]. The Gunn rat, a mutant of the Wistar strain [23], has a genetic deficiency in glucuronyl transferase and has been used as an animal model of bilirubin encephalopathy. We found that a high serum UCB concentration has a pathogenic effect on development of the brain and concluded that the Gunn rat may be used as an animal model of schizophrenia [22].

UCB in the CNS is toxic to neurons and associated microglia, the resident immune cells in the CNS [24-26]. However, the effects of UCB on microglia in Gunn rats have never been investigated. Therefore, in the present study, we sought to examine how microglial cells respond to UCB toxicity in Gunn rats. We hypothesized that UCB toxicity induces microglia activation and that prolonged microglial activation plays a role that makes the Gunn rat suitable as an animal model of schizophrenia. We observed the morphological features, distribution, and ultrastructural characteristics of microglial cells in adult Gunn rats. We also determined the ratio of resting/ramified cells to activated cells and examined the neuron-microglia interactions. These studies were performed on the hippocampal dentate gyrus (DG), and the results were compared to those to Wistar rats as a normal control.

## Methods

### Animals

The animals were male homozygous (j/j) Gunn rats and male Wistar rats (N = 10 each, Japan SLC, Inc., Shizuoka, Japan) that were 8 weeks old at the time of the experiments. Gunn j/j rats were born to female Gunn j/j and male +/j rats at Japan SLC, Inc. The rats were housed under standard conditions with a room temperature of 23 ± 2°C, humidity of 55 ± 5%, and 12 h light, 12 h dark cycle (light phase 7:00 to 19:00) and were given free access to food and water. Starting 1 week before the beginning of the experiment, the rats underwent a handling procedure once that was aimed at reducing experimental stress. The handling procedure consisted of picking the rat up with a gloved hand and stroking it for 10 minutes. All procedures were performed with the approval of the Shimane University Animal Ethics Committee, under the guidelines of the National Health and Medical Research Council of Japan.

Under deep intraperitoneal anesthesia with sodium pentobarbital (80 mg/kg body weight), the rats were perfused transcardially with 500 ml of physiological saline, followed by 500 ml of 4% paraformaldehyde in 0.1 M phosphate buffer (PB; pH 7.3). After perfusion, the brain was quickly removed, post fixed in a solution of 4% paraformaldehyde at room temperature for 4 h, and then immersed overnight in a cold solution of 20% sucrose. Later, the brains were cut in at 50 μm thick in the frontal plane using a freezing microtome.

### Immunohistochemistry for light and confocal laser scanning microscopy

Sections used for immunohistochemistry were incubated in 1% H_2_O_2 _for 30 minutes, then were rinsed twice for 15 minutes in PBS. Free-floating sections were preincubated with 1.5% normal goat serum, 0.2% Triton-X in 0.1 M PB (pH 7.3) for 1 h rotating at room temperature (RT). Sections were then incubated overnight rotating at RT with primary antibody in preincubation solution. The following primary antibodies were used: rabbit anti-ionized calcium binding adaptor molecule 1 (Iba1) (1:4,000, Wako Ltd., Osaka, Japan) and mouse anti-CD11b (1:500, AbD Serotec, Oxford, UK). The sections were then rinsed twice for 15 minutes in phosphate-buffered saline (PBS) and incubated for 1 h in biotinylated anti-rabbit (or anti-mouse) IgG (1:200, Standard ABC Kit, Vector Labs, Burlingame, CA, USA) solution, rotating at RT. The sections were then rinsed twice for 15 minutes in PBS and incubated for 1 h in PBS containing avidin-biotin peroxidase complex (ABC) (Standard ABC Kit, Vector Labs) solution rotating at RT. After being rinsed twice for 15 minutes in PBS, the sections were developed by incubating in PBS containing 10 mg diaminobenzidine (DAB) and 5 μl of 30% hydrogen peroxide for 10 minutes. The DAB reaction was halted using PBS, followed by two 15-minute PBS rinses. The tissue was mounted onto gelatin-coated slides, dehydrated in graded alcohol baths, and coverslips (Matsunami Glass Ind., Ltd., Osaka, Japan) were applied using mounting medium. For Nissl staining, the sections were stained with 0.5% cresyl violet, dehydrated, delipidated in xylene, and coverslipped. For double immunofluorescent labeling, sections were incubated free floating in secondary antibodies: mouse anti-CD11b (1:500, Serotec), or mouse anti-NeuN (neuronal nuclei) antibody (1:200, Millipore Inc., Temecula, CA, USA) with 1.5% normal goat serum, 0.2% Triton-X in 0.1 M PB overnight rotating at RT. Subsequently, the sections were rinsed twice for 15 minutes in PBS, then were incubated in PBS containing Cy3-conjugated anti-rabbit IgG for Iba1 (Amersham Bioscience Ltd., Piscataway, NJ, USA) diluted at 1:1,000 and Alexa488-conjugated anti-mouse IgG for CD11b (Invitrogen, Carlsbad, CA, USA) diluted at 1:1,000 for 1 h.

### Iba1-immunoelectron microscopy

Rats were perfused transcardially with a solution composed of 4% paraformaldehyde and 0.1% glutaraldehyde in 0.1 M PB (pH 7.3), and the brain was quickly removed, post fixed in a solution of 4% paraformaldehyde at room temperature for 4 h, and then immersed overnight in a cold solution of 20% sucrose in 0.1 M PB (pH 7.3). Then, the hippocampus area was dissected out and cut at 50 μm thickness in the frontal plane using a vibratome microtome. The sections were incubated overnight with rabbit anti-Iba1 (1:4,000, Wako) in preincubation solution rotating at RT. After being rinsed in PBS, the sections were incubated for 1 h in biotinylated anti-rabbit IgG (1:200), Vector Labs), rinsed in PBS, and then incubated for 1 h in PBS containing ABC (Standard ABC Kit, Vector Labs). The sections were then rinsed in PBS and incubated in PBS containing 10 mg DAB and 5 μl of 30% hydrogen peroxide. Later the sections were post fixed in a solution of 1% osmium tetroxide in 0.1 M PB (pH 7.3) for 30 minutes at RT. After washing in distilled water, the sections were stained en bloc with 0.5% uranyl acetate in 70% ethanol for 1 h, dehydrated in a graded series of ethanols, cleared in propylene oxide, and then embedded flat in Epon. Subsequently, serial ultrathin sections were cut on an ultramicrotome, collected on collodion-coated copper grids, and stained with lead citrate. Finally, the sections were examined under an electron microscope (JEOL JEM1200EX).

### Counting Iba1-labeled microglial cells in DG

To estimate the numbers of Iba1 expressing microglial cells, images were captured from four areas within the hippocampal DG: hilus, subgranular zone (SGZ), granular layer (GL), and molecular layer (ML). A 55 × 55 μm box was then randomly placed within each region of interest from interaural 5.88 mm, bregma -3.12 mm, interaural 4.20 mm, and bregma -4.80 mm, based on the atlas of the rat brain [27]. There were about 450 boxes per region in each sample at each analysis. The images were analyzed using Stereo Investigator software V. 7.0 (MicroBrightfield Inc., Williston, VT, USA). The software automatically calculates the number of Iba1-labeled cells within the region of interest.

### Measuring CD11b-labeled microglial cells immunoreactive area

The immunoreactive area was measured using a computer-assisted image analysis program (Image Tool V. 3.0, Department of Dental Diagnostic Science, University of Texas Health Science Center, San Antonio, Texas, USA). Sections containing CD11b-labeled cells were examined under a light microscope (Nikon, ECLIPSE E800). Images (N = 40 per region examined, per time point) were randomly captured from the same region of interest used in the cell counting procedure, and these areas were used for analysis. The images were analyzed using the ImageTool software, which automates the analysis by converting all immunolabeled elements that fall within a threshold range into pure black pixels and the rest of the image into pure white pixels. The software then quantifies the total number and percentages of black and white pixels, allowing for statistical analysis of the data.

### Statistical analysis

Statistical analysis was performed with SPSS software (Dr. SPSS II for Windows V. 11.0, SPSS Inc., Chicago. IL, USA) and the results were presented as the mean ± SEM. Differences between Gunn rats and controls were compared using the two-tailed Student's t test with *P *of < 0.005 considered to be a significant difference.

## Results

### Light and confocal microscopy of Iba1-labeled microglial cells

**Figure 1:**
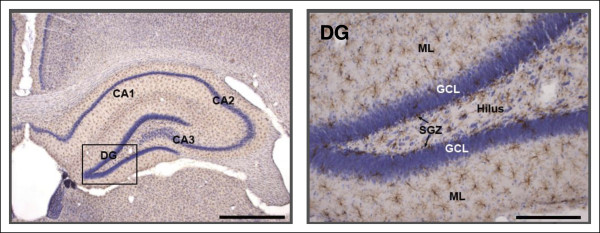
**Hippocampal dentate gyrus (DG) of Gunn rat**. Immunolabeling using ionized calcium binding adaptor molecule 1 (Iba1)/Nissl staining. Scale bars: 0.5 mm (left); 0.1 mm (right).

**Figure 2:**
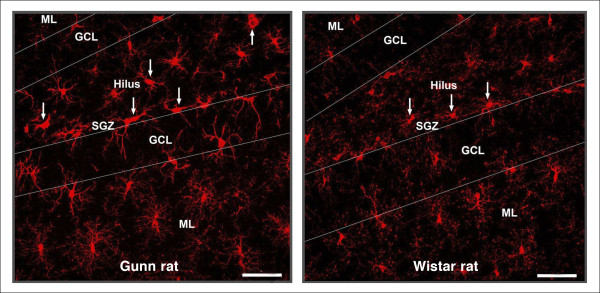
**Confocal photomicrographs of ionized calcium binding adaptor molecule 1 (Iba1)-labeled microglial cells in dentate gyrus (DG)**. A confocal Z-stack merged image depicting Iba1-labeled cell bodies (red), processes, and distribution in the DG in Gunn rats **(A) **and Wistar rats as a normal control **(B)**. Immunolabeling using Iba1/NeuN staining. Scale bars: 50 μm.

We found Iba1-labeled microglial cells throughout the hilus and in the SGZ, GL, and ML in the hippocampal DG (Figure 1). Iba1-labeled cells in Gunn rats showed elongated cell somata with fewer branches but thicker processes in the hilus, SGZ, and GL (Figure 2A). This kind of morphology is known as activated morphology [28]. However, in the ML cells showed small rod-shaped somata with numerous thin and highly ramified processes. This kind of morphology is known as ramified/resting morphology [29] and is similar to the morphology found in Wistar rats (Figure 2B). Iba1-labeled cells in the GL were relatively infrequent both in Gunn and Wistar rats. However, a concentration of Iba1-labeled cells was found at the SGZ with processes extending into the GL.

**Figure 3:**
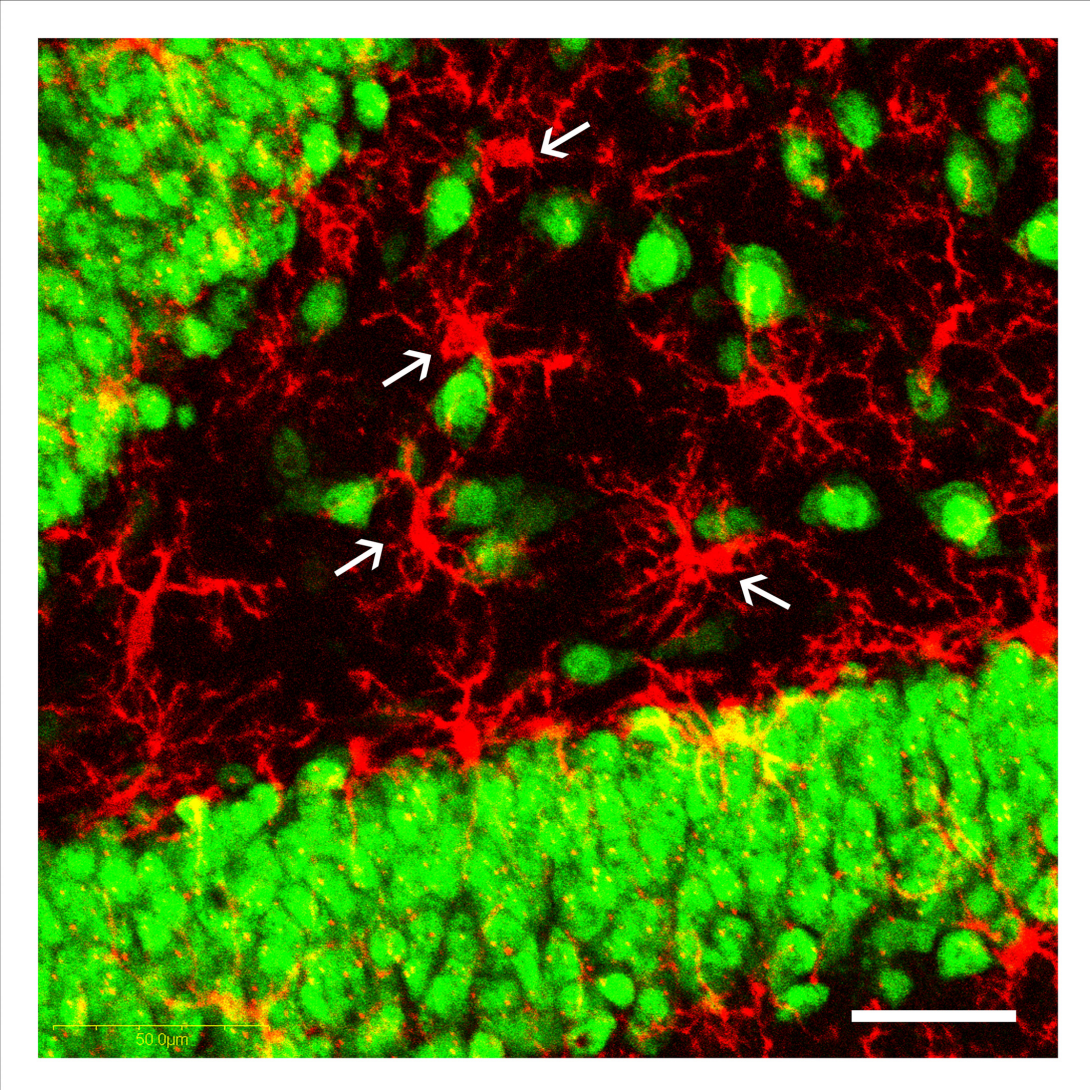
**Microglial-neuron interaction**. A confocal Z-stack merged image in the hilus of a Gunn rat demonstrates apposition of microglial cells and neuronal somata. Immunolabeling using ionized calcium binding adaptor molecule 1 (Iba1)/NeuN staining. Scale bars: 50 μm.

Some Iba1-labeled cells in Gunn rats were observed in apposition to NeuN-labeled neuronal cell bodies (Figure 3). These Iba1 cells showed morphology of the activated type with processes that try to wrap around the neuron cell body.

**Figure 4:**
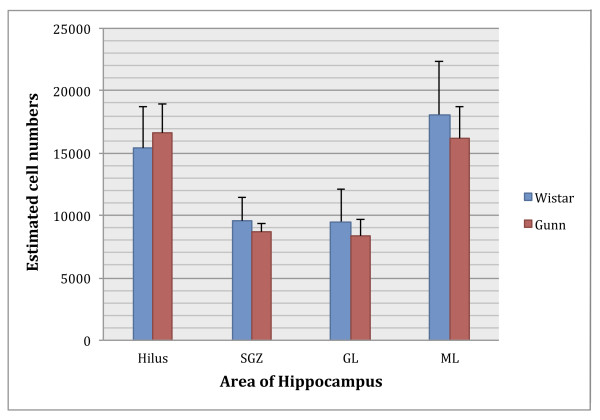
**Estimated ionized calcium binding adaptor molecule 1 (Iba1)-labeled microglial cell numbers in dentate gyrus (DG)**. A two-tailed t test did not reveal any significant difference between Gunn and Wistar rats in any DG samples. Data are presented as mean ± SEM. Area of hippocampus: hilus; ML = molecular layer; GL = granular layer; SGZ = subgranular zone.

Then we counted the Iba1-labeled cell numbers using the Stereo Investigator software. We captured about 450 sites, and counted for approximately 2 to 3 Iba1-labeled cells per site. The software then automatically estimated automated the cell numbers. Our result showed no significant difference between Gunn and Wistar rats in the estimated numbers of Iba1-labeled cells in all areas of the DG (Figure 4).

### Electron microscopy of Iba1-labeled microglial cells

**Figure 5:**
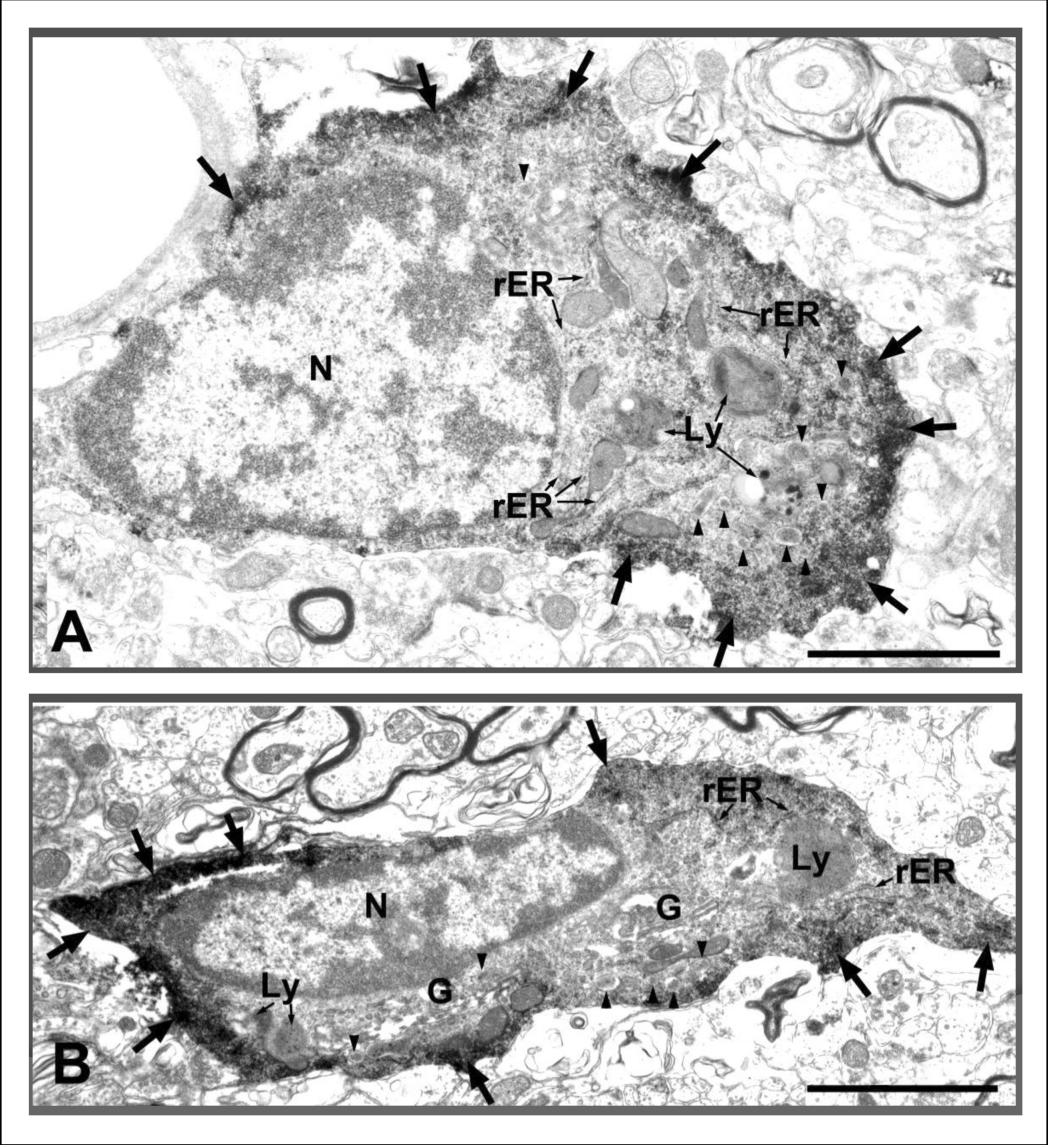
**Electron micrographs of ionized calcium binding adaptor molecule 1 (Iba1)-labeled microglial cells in the hilus area of Gunn rat**. Arrows represent the increased electron density at the periphery of cytoplasm; arrowheads represent small vesicles containing low-density material. Scale bars: 2 μm. G = Golgi apparatus; Ly = lysosome; N = nucleus; rER = rough endoplasmic reticulum.

Ultrastructurally, Iba1-labeled cell bodies in the DG were identified by an electron-dense immunoreactive product within their perikaryal cytoplasm [30]. Activated Iba1-labeled cells have an elongated cell body and enlarged cytoplasm (Figure 5). There was an increased electron density in the periphery of the cytoplasm. We also observed an organelle-rich cytoplasm that includes the Golgi apparatus, lysosomes, rough endoplasmic reticulum, and some small vesicles containing low-density material.

**Figure 6:**
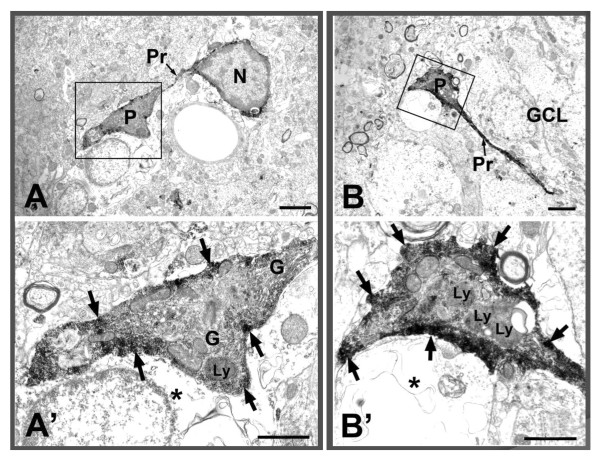
**ionized calcium binding adaptor molecule 1 (Iba1)-labeled microglial cells with phagocytic pouch in Gunn rat**. Somata of Iba1-labeled cells with extended processes and phagocytic pouch attached to damaged/broken cells (*). Arrows indicate immunoreactive products. Notice the plentiful organelles within the pouch. Scale bars: 2 μm (A, B), 1 μm (A', B'). G = Golgi apparatus; GCL = granular cell layer; Ly = lysosome; N = nucleus; P = phagocytic pouch; Pr = process.

**Figure 7:**
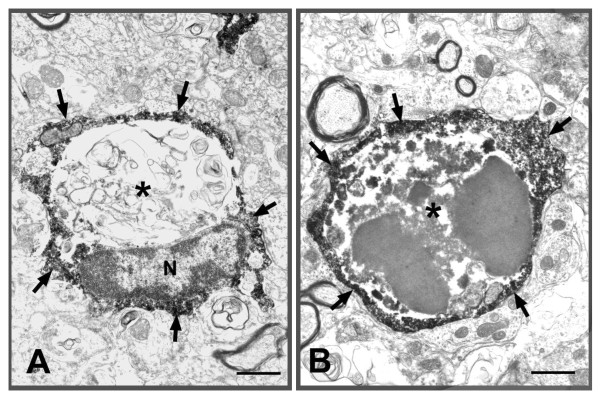
**Electron micrographs of ionized calcium binding adaptor molecule 1 (Iba1)-labeled macrophage-like cells in Gunn rat**. Two examples of macrophage-like cells with large phagocytic vacuole/vesicle (*). Arrows indicate the immunoreactive products. Scale bars: 1 μm.

Another interesting finding was the phagocytic function of microglia. We show two examples of microglial cells with the processes and phagocytic pouches formed at the ends of the processes (Figure 6). This kind of phagocytic pouch is known as a modification of the phagocytosis function of microglial processes [31]. These phagocytic pouches seemed to attach to damaged or broken cells. There were rich organelles inside the pouches, and strong immunoreaction products were observed in the peripheral cytoplasm (Figure 6A, B). Finally, macrophage-like microglial cells were observed in the dentate gyrus of Gunn rats. Two examples of Iba1-labeled macrophage-like cells with large phagocytic vacuoles/vesicles are shown in Figure 7. We can still observe the immunoreactive product around the cells, but no more organelle-rich cytoplasm is seen.

### CD11b expression in Iba1-labeled microglial cells

**Figure 8:**
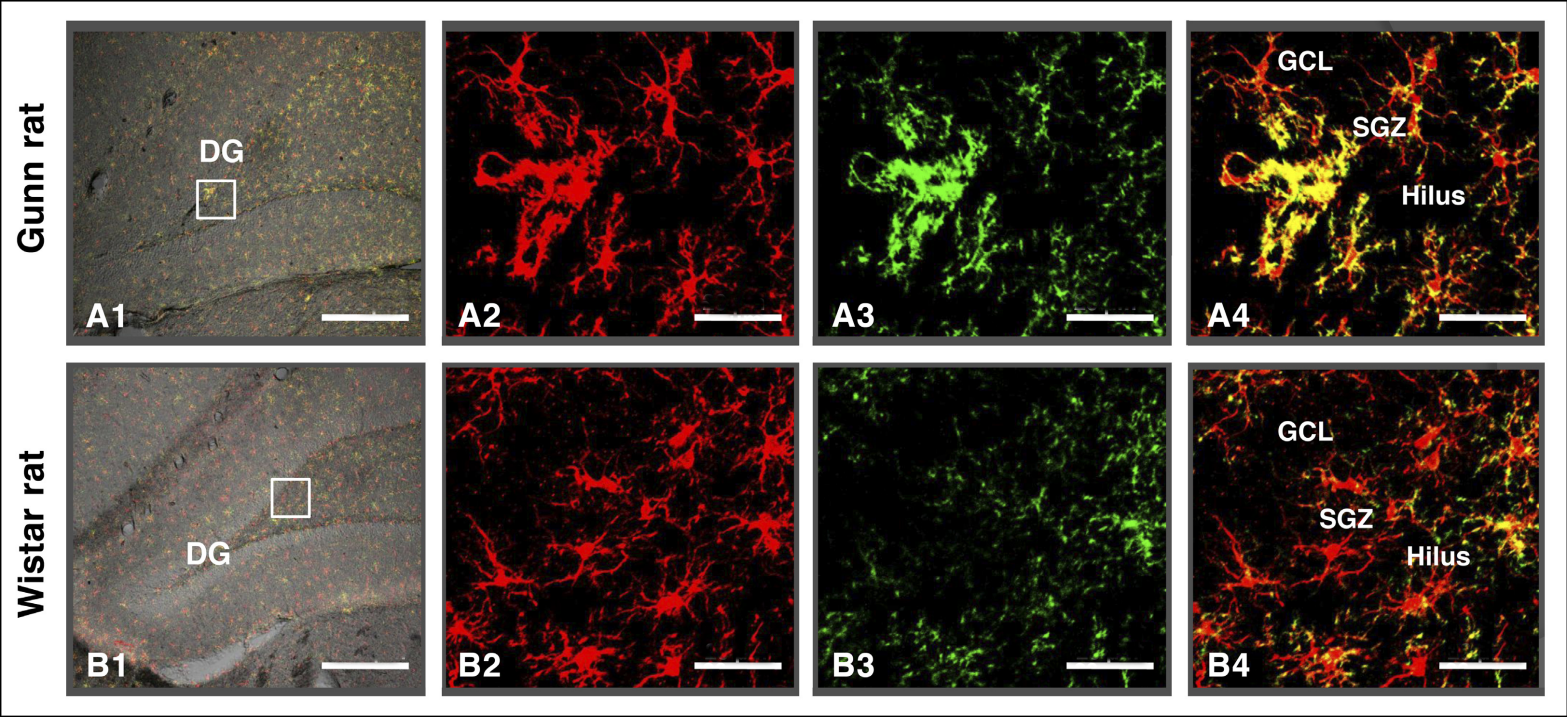
**CD11b expression in ionized calcium binding adaptor molecule 1 (Iba1)-labeled microglial cells**. A confocal Z stack image depicting Iba1-labeled (red), CD11b expression (green), and merged (yellow) cells of a Gunn rat **(A1-A4) **compared to those of a Wistar rat **(B1-B4) **in the hippocampal dentate gyrus (DG). Microglial cells in Gunn rats expressed a high level of CD11b immunoreactivity compared to those of Wistar rats. Scale bars: 200 μm (A1, B1), 20 μm (A2-A4, B2-B4).

Next, we examined the expression of a marker that increases during microglia activation, CD11b (integrin α M) [31]. The expression of CD11b in Iba1-labeled microglial cells in the DG of Gunn rats was compared to its expression in Wistar rats as normal controls (Figure 8). Our result showed that microglial cells in Gunn rats expressed high levels of CD11b immunoreactivity (Figure 8A3, 8A4). Interestingly, some of the positive CD11b microglial cells were bunched together in the SGZ. In contrast, microglial cells in Wistar rats did not show significant CD11b expression (Figure 8B3, 8B4).

**Figure 9:**
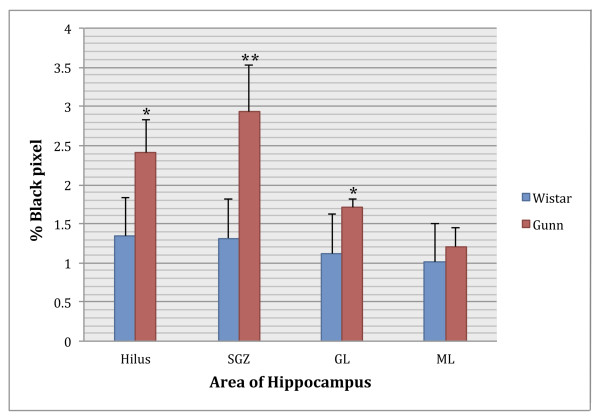
**Mean percentage of black pixels indicating CD11b expression area in the dentate gyrus (DG)**. A two-tailed t test revealed that there were significantly larger areas of CD11b expression in Gunn rats than in Wistar rats. *P ≤ 0.005, **P ≤ 0.001, vs Wistar. Data are presented as mean ± SEM. Area of hippocampus: hilus; GL = granular layer; ML = molecular layer; SGZ = subgranular zone.

Furthermore, we determined the mean percentages of the CD11b expression areas in the hilus, SGZ, GL, and ML of DG. Statistical analysis showed that areas of CD11b expression were significantly greater in Gunn rats than in Wistar rats in the hilus (*P *< 0.005), SGZ (*P *< 0.001), and GL (*P *< 0.005). There were no significant difference of CD11b expression in the ML between Gunn rats and Wistar rats (Figure 9).

## Discussion

In the present study, we demonstrated the morphological features of microglial cells in the DG of Gunn rats for the first time. We used immunofluorescent labeling with an antibody against Iba1 to identify microglia, and CD11b to observe phagocytic microglial activation [31].

First, we revealed that Iba1-labeled microglial cells showed activated morphology in the DG of Gunn rats. Second, during ultrastructural observation, we found that these activated cells contained enlarged areas of cytoplasm rich in organelles, and that some of them formed phagocytic pouches or engulfed large phagocytic vacuoles. Third, there was significant difference in CD11b expression areas in the DG of Gunn rats compared to controls.

Microglial cells are generally considered to be the immune cells of the CNS and perform the function of monitoring and protecting the well-being of neurons [32,33]. Under normal conditions, the number of microglia is limited, comprising 20% of the total glial cell population in the brain, and are characterized by a small cell body with fine, ramified processes, and low expression of surface antigens [33,34]. When the CNS is injured, microglia rapidly shift into an activated state and migrate to the damaged sites. Activated microglia are marked by a number of characteristic events, affecting cellular morphology, cell size, cell number, and at the molecular level, the pattern of cell surface molecules expressed (immunophenotype) as well as the pattern of cytokines and growth factors produced, which distinguish them from the resting/ramified phenotype [34,35].

Microglial cells are well known to interact with neurons. Microglia are able to regulate several aspects of neuronal functions, and neurons can control microglia activation [36]. Microglial cells respond to signals from injured neurons, which shift them into activated states, in close apposition to the injured neuron in order to release either neurotropic or neurotoxic factors [35]. Our result found that some microglial cells were apposed to neurons and showed activated morphology. We suggest that these neurons probably were injured by UCB toxicity, and therefore induced microglia activation.

An additional highly characteristic feature of microglia activation is the remarkable capacity of the microglial cell population to expand, especially in response to acute injury [37]. This expansion is usually transient and has been considered to be due predominantly to proliferation of activated resident microglia [35,38]. Our result showed no significant difference between Iba1-labeled microglial cell numbers in Gunn rats and in controls. However, we found a significant difference in the area of CD11b expression as marker of microglial activation. These results suggest that microglial cells in adult Gunn rats showed a feature of microglial activation without expansion of the cell population.

Homozygous (j/j) Gunn rats have an inherited deficiency of hepatic UDP-glucuronyl transferase and therefore are unable to adequately conjugate and excrete bilirubin. This condition develops into severe hyperbilirubinemia, with visible jaundice within 6 h of parturition, and thus manifests a pattern of brain injury that resembles the histopathology of neuronal injury associated with bilirubin encephalopathy in humans. This bilirubin neurotoxicity may persist through their entire lives [39,40]. In homozygous (j/j) Gunn rats, few signs of bilirubin toxicity are present during the first postnatal weeks [41]. Our previous study found that blood bilirubin levels in adult Gunn rats were still high [22] and the presence of microglia activation suggested the possibility of chronic neuronal inflammation. When acute microglial activation becomes a chronic condition following injury, the microglial cells is potentially maladaptive or neuroprotective [42]. We suggest that chronic microglial activation in adult Gunn rats is potentially more maladaptive than neuroprotective.

Prolonged microglia activation of microglia may lead to neuronal degeneration, white matter abnormalities, decreased neurogenesis, apoptosis, and brain damage, and may thus be one of the important factors in the pathophysiology of schizophrenia [2]. Moreover, a previous positron emission tomography study showed significant neuroinflammation in the hippocampus of schizophrenic patients compared with healthy volunteers [43]. Increased serum concentrations of proinflammatory cytokines have been observed in schizophrenia patients [44,45]. In clinical practice, immunomodulatory drugs such as cyclo-oxygenase 2 and minocycline have been reported to have beneficial effects on schizophrenia treatment [46,47]. Moreover, it was reported that atypical antipsychotics may have anti-inflammatory effects on microglial activation [48,49].

## Conclusions

In summary, our results showed evidence of microglial activation within the brains of adult Gunn rats. We suggest that the chronic microglial activation due to UCB toxicity could be considered an important causal factor in the behavioral abnormalities and neuropathological changes in Gunn rats. Moreover, our result may provide crucial information to elucidate the etiology of schizophrenia and support the possibility of using Gunn rats as an animal model of schizophrenia. However, the exact mechanism of chronic microglial activation in Gunn rats is still unclear. Future studies are needed to elucidate the mechanism of UCB toxicity in neurons and microglia.

## Competing interests

The authors declare that they have no competing interests.

## Authors' contributions

Conception and design of experiments: TM, KL, RW, MF, MI. Immunohistochemistry, analysis, and interpretation of data: KL, TT, KT, MT, KI. Electron microscopy data: KL, TT, KT. Writing and/or critical review of the article: KL, TM, TT, MF, AJT, JH. All authors read and approved the final manuscript.

## References

[B1] Saha S Chant D Welham J McGrath J A systematic review of the prevalence of schizophrenia PLoS Med 2005 2 413 433 10.1371/journal.pmed.0020141 PMC1140952 15916472

[B2] Monji A Kato T Kanba S Cytokines and schizophrenia: microglia hypothesis of schizophrenia Psychiatry Clin Neurosci 2009 63 257 265 10.1111/j.1440-1819.2009.01945.x 19579286

[B3] Stefansson H Ophoff RA Steinberg S Andreassen OA Cichon S Rujescu D Werge T Pietiläinen OP Mors O Mortensen PB Sigurdsson E Gustafsson O Nyegaard M Tuulio-Henriksson A Ingason A Hansen T Suvisaari J Lonnqvist J Paunio T Børglum AD Hartmann A Fink-Jensen A Nordentoft M Hougaard D Norgaard-Pedersen B Böttcher Y Olesen J Breuer R Möller HJ Giegling I Common variants conferring risk of schizophrenia Nature 2009 460 744 748 19571808 10.1038/nature08186 PMC3077530

[B4] Miller BJ Buckley P Seabott W Mellor A Kirkpatrick B Meta-analysis of cytokine alterations in schizophrenia: clinical studies and antipsychotic effects Biol Psychiatry 2011 70 663 671 10.1016/j.biopsych.2011.04.013 21641581 PMC4071300

[B5] Drexhage RC Knijff EM Padmos RC Heul-Nieuwenhuijzen L Beumer W Versnel MA Drexhage HA The mononuclear phagocyte system and its cytokine inflammatory networks in schizophrenia and bipolar disorder Expert Rev Neurother 2010 10 59 76 10.1586/ern.09.144 20021321

[B6] Steiner J Mawrin C Ziegeler A Bielau H Ullrich O Bernstein HG Bogerts B Distribution of HLA-DR-positive microglia in schizophrenia reflects impaired cerebral lateralization Acta Neuropathol 2006 112 305 316 10.1007/s00401-006-0090-8 16783554

[B7] Steiner J Bielau H Brisch R Danos P Ullrich O Mawrin C Bernstein HG Bogerts B Immunological aspects in the neurobiology of suicide: elevated microglial density in schizophrenia and depression is associated with suicide J Psychiatr Res 2008 42 151 157 10.1016/j.jpsychires.2006.10.013 17174336

[B8] Muller N Myint AM Schwarz MJ Kynurenine pathway in schizophrenia: pathophysiological and therapeutic aspects Curr Pharm Des 2011 17 130 136 10.2174/138161211795049552 21361867

[B9] Steiner J Bogerts B Sarnyai Z Walter M Gos T Bernstein HG Myint AM Bridging the gap between the immune and glutamate hypotheses of schizophrenia and major depression: potential role of glial NMDA receptor modulators and impaired blood-brain barrier integrity World J Biol Psychiatry in press 10.3109/15622975.2011.583941 21707463

[B10] Muller N Schiller P Ackenheil M Coincidence of schizophrenia and hyperbilirubinemia Pharmacopsychiatry 1991 24 225 228 10.1055/s-2007-1014472 1812499

[B11] Miyaoka T Seno H Itoga M Iijima M Inagaki T Horiguchi J Schizophrenia-associated idiopathic unconjugated bilirubinemia (Gilbert's syndrome) J Clin Psychiatry 2000 61 868 871 10.4088/JCP.v61n1110 11105741

[B12] Radhakrishnan R Kanigere M Menon J Calvin S Janish A Srinivasan K Association between unconjugated bilirubin and schizophrenia Psychiatry Res 2011 189 480 482 10.1016/j.psychres.2011.03.003 21470692

[B13] Miyaoka T Yasukawa R Mizuno S Sukegawa T Inagaki T Horiguchi J Seno H Oda K Kitagaki H Proton magnetic resonance spectroscopy (1H-MRS) of hippocampus, basal ganglia, and vermis of cerebellum in schizophrenia associated with idiopathic unconjugated hyperbilirubinemia (Gilbert's syndrome) J Psychiatr Res 2005 39 29 34 10.1016/j.jpsychires.2004.05.003 15504421

[B14] Yasukawa R Miyaoka T Mizuno S Inagaki T Horiguchi J Oda K Kitagaki H Proton magnetic resonance spectroscopy of the anterior cingulated gyrus, insular cortex and thalamus in schizophrenia associated with idiopathic unconjugated hyperbilirubinemia (Gilbert's syndrome) J Psychiatry Neurosci 2005 30 416 422 16327875 PMC1277024

[B15] Miyaoka T Yasukawa R Mihara T Mizuno S Yasuda H Sukegawa T Hayashida M Inagaki T Horiguchi J Fluid-attenuated inversion-recovery MR imaging in schizophrenia-associated with idiopathic unconjugated hyperbilirubinemia (Gilbert's syndrome) Eur Psychiatry 2005 20 327 331 10.1016/j.eurpsy.2004.12.012 16018925

[B16] Wake R Miyaoka T Tsuchie K Kawakami K Nishida A Inagaki T Horiguchi J Abnormalities in MRI signal intensity in schizophrenia associated with idiopathic unconjugated hyperbilirubinemia Aus N Z J Psychiatry 2009 43 1057 1069 10.1080/00048670903107526 20001401

[B17] Dennery PA Seidman DS Stevenson DK Neonatal hyperbilirubinemia N Engl J Med 2001 344 581 590 10.1056/NEJM200102223440807 11207355

[B18] Hansen TWR Mechanism of bilirubin toxicity: clinical implications Clin Perinatol 2002 29 765 778 10.1016/S0095-5108(02)00053-2 12516745

[B19] Porter ML Dennis BL Hyperbilirubinemia in the term newborn Am Fam Physician 2002 65 599 606 11871676

[B20] Shapiro SM Definition of the clinical spectrum of kernicterus and bilirubin-induced neurologic dysfunction (BIND) J Perinatol 2005 25 54 59 10.1038/sj.jp.7211157 15578034

[B21] Dalman C Cullberg J Neonatal hyperbilirubinemia-vulnerability factor for mental disorder? Acta Psychiatr Scand 1999 100 469 471 10.1111/j.1600-0447.1999.tb10899.x 10626927

[B22] Hayashida M Miyaoka T Tsuchie K Yasuda H Wake R Nishida A Inagaki T Toga T Hagami H Oda T Horiguchi J Hyperbilirubinemia-related behavioral and neuropathological changes in rats: a possible schizophrenia animal model Prog Neuropsychopharmacol Biol Psychiatry 2009 33 581 588 10.1016/j.pnpbp.2009.02.013 19249333

[B23] Gunn CK Hereditary acholuric jaundice in the rat Can Med Assoc J 1944 50 230 237 20323028 PMC1581738

[B24] Silva RFM Rodriques CM Brites D Rat cultured neuronal and glial cells respond differently to toxicity of unconjugated bilirubin Pediatr Res 2002 51 535 541 10.1203/00006450-200204000-00022 11919342

[B25] Gordo AC Falcão AS Fernandes A Brito MA Slva RFM Brites D Unconjugated bilirubin activates and damages microglia J Neurosci Res 2006 84 194 201 10.1002/jnr.20857 16612833

[B26] Silva SL Vaz AR Barateiro A Falcão AS Fernandes A Brito MA Silva RFM Brites D Features of bilirubin-induced reactive microglia: from phagocytosis to inflammation Neurobiol Dis 2010 40 663 675 10.1016/j.nbd.2010.08.010 20727973

[B27] Paxinos G Watson C The Rat Brain in Stereotaxic Coordinates 2007 6 London: Elsevier Inc

[B28] Streit WJ Graeber MB Kreutzberg GW Functional plasticity of microglia: a review Glia 1988 1 301 307 10.1002/glia.440010502 2976393

[B29] Nimmerjahn A Kirchhcoff F Helmchen F Resting microglia cells are highly dynamic surveillants of brain parenchyma in vivo Science 2005 308 1314 1318 10.1126/science.1110647 15831717

[B30] Shapiro LA Perez ZD Foresti ML Arisi GM Ribak CE Morphological and ultrastructural features of Iba1-immunolabeled microglial cells in the hippocampal dentate gyrus Brain Res 2009 1266 29 36 19249294 10.1016/j.brainres.2009.02.031 PMC2677570

[B31] Sierra A Encinas JM Deudero JJP Chancey JH Enikolopov G Overstreet-Wadiche LS Tsirka SE Maletic-Savatic M Microglia shape adult hippocampal neurogenesis through apoptosis-coupled phagocytosis Cell Stem Cell 2010 7 483 495 10.1016/j.stem.2010.08.014 20887954 PMC4008496

[B32] Rossum VD Hanisch UK Microglia Metab Brain Dis 2004 19 393 411 15554430 10.1023/b:mebr.0000043984.73063.d8

[B33] Hanisch UK Kettenmann H Microglia: active sensor and versatile effector cells in the normal and pathologic brain Nat Neurosci 2007 10 1387 1394 10.1038/nn1997 17965659

[B34] Graeber MB Changing face of microglia Science 2010 330 783 788 10.1126/science.1190929 21051630

[B35] Streit WJ Walter SA Pennel NA Reactive microgliosis Prog Neurobiol 1999 57 563 581 10.1016/S0301-0082(98)00069-0 10221782

[B36] Bessis A Béchade C Bernard D Roumier A Microglial control of neuronal death and synaptic properties Glia 2007 55 233 238 10.1002/glia.20459 17106878

[B37] Ladeby R Wirenfeldt M Garcia-Overejo D Fenger C Dissing Olsen L Dalmau I Finsen B Microglial cell population dynamics in the injured adult central nervous system Brain Res Rev 2005 48 196 206 10.1016/j.brainresrev.2004.12.009 15850658

[B38] Hailer NP Grampp A Nitsch R Proliferation of microglia and astrocytes in the dentate gyrus following entohirnal cortex lesion: a quantitative bromodeoxyuridine-labelling study Eur J Neurosci 1999 11 3359 3364 10.1046/j.1460-9568.1999.00808.x 10510203

[B39] Ahdab-Barmada M Moossy J The neuropathology of kernicterus in the premature neonate: diagnostic problems J Neuropathol Exp Neurol 1984 43 45 52 10.1097/00005072-198401000-00004 6693927

[B40] Johnson L Sarmiento F Blanc WA Day R Kernicterus in rats with inherited deficiency of glucuronyl transferase AMA J Dis Child 1959 97 591 608 13649090 10.1001/archpedi.1959.02070010593009

[B41] McDonald JW Shapiro SM Silverstein FS Johnston MV Role of glutamate receptor-mediated excitotoxicity in bilirubin-induced brain injury in the Gunn rat model Exp Neurol 1998 150 21 29 10.1006/exnr.1997.6762 9514835

[B42] Ekdahl CT Kokaia Z Lindvall O Brain inflammation and adult neurogenesis: the dual role of microglia Neuroscience 2009 158 1021 1029 10.1016/j.neuroscience.2008.06.052 18662748

[B43] Doorduin J de Vries EFJ Willemsen ATM de Groot JC Dierckx RA Klein HC Neuroinflammation in schizophrenia-related psychosis: a PET study J Nucl Med 2009 50 1801 1807 10.2967/jnumed.109.066647 19837763

[B44] Lin A Kenis G Bignotti S Tura GJ de Jong R Bosmans E Pioli R Altamura C Scharpé S Maes M The inflammatory response system in treatment-resistant schizophrenia: increased serum interleukin-6 Schizophr Res 1998 32 9 15 10.1016/S0920-9964(98)00034-6 9690329

[B45] Zhang XY Zhou DF Cao LY Zhang PY Wu GY Shen YC Changes in serum interleukin-2, -6, and -8 levels before and during treatment with risperidone and haloperidol: relationship to outcome in schizophrenia J Clin Psychiatry 2004 65 940 947 10.4088/JCP.v65n0710 15291683

[B46] Akhondzadeh S Tabatabaee M Amini H Ahmadi Abhari SA Abbasi SH Behnam B Celecoxib as adjunctive therapy in schizophrenia: a double-blind, randomized, and placebo-controlled trial Schizophr Res 2007 90 179 185 10.1016/j.schres.2006.11.016 17208413

[B47] Miyaoka T Yasukawa R Yasuda H Hayashida M Inagaki T Horiguchi J Minocycline as adjunctive therapy for schizophrenia: an open-label study Clin Neuropharmacol 2008 315 287 292 18836347 10.1097/WNF.0b013e3181593d45

[B48] Kato T Monji A Hashioka S Kanba S Risperidone significantly inhibits interferon-gamma-induced microglial activation in vitro Schizophr Res 2007 92 108 115 10.1016/j.schres.2007.01.019 17363222

[B49] Bian Q Kato T Monji A Hashioka S Mizoguchi Y Horikawa H Kanba S The effect of atypical antipsychotics, perospirone, ziprasidone, and quetiapine on microglial activation induced by interferon-gamma Prog Neuropsychopharmacol Biol Psychiatry 2008 32 42 48 10.1016/j.pnpbp.2007.06.031 17716796

